# Evaluation of Nurses’ Vaccine Hesitancy, Psychological Resilience, and Anxiety Levels During COVID-19 Pandemic

**DOI:** 10.5152/eurasianjmed.2023.22162

**Published:** 2023-06-01

**Authors:** Sami Akbulut, Gülseda Boz, Ayşe Gökçe, Selver Ünsal, Hasan Sarıtaş, Erva Kızılay, Ali Özer, Mehmet Serdar Akbulut, Cemil Çolak

**Affiliations:** 1 Department of Surgery and Liver Transplant Institute, Inonu University, Faculty of Medicine, Malatya, Turkey; 2 Department of Public Health, Inonu University, Faculty of Medicine, Malatya, Turkey; 3 Department of Biostatistics, and Medical Informatics, Inonu University, Faculty of Medicine, Malatya, Turkey; 4 Department of Nursing Service, Inonu University, Faculty of Medicine, Malatya, Turkey; 5 Department of Surgical Nursing, Siirt University, Faculty of Health Science, Siirt, Turkey; 6 Department of Social Work, Bingol State Hospital, Bingol, Turkey

**Keywords:** COVID-19 pandemic, nurses, vaccine hesitancy, psychological resilience, anxiety

## Abstract

**Objective:**

This study aimed to evaluate the vaccine hesitancy, psychological resilience, and anxiety levels of nurses during the COVID-19 pandemic.

**Materials and Methods:**

This cross-sectional study was conducted with 676 nurses working at the survey time. Sociodemographic features, the status of hesitancy against the COVID-19 vaccine, the Coronavirus Anxiety Scale, and the Brief Resilience Scale were used in the questionnaire form to collect the data.

**Results:**

Most participants (68.6%; n = 464) stated they were hesitant about the COVID-19 vaccine. A significantly higher rate of hesitancy was detected in the age group of 20-39 years, those who did not have COVID-19 vaccine, and those who did not think the COVID-19 vaccine is protective (*P < .*05). It was determined that 6.8% (n = 46) of the nurses had COVID-19 anxiety. A significantly higher rate of anxiety was detected in the age group of 40 years and older, those working in the emergency department, and those working in the COVID-19 unit during the pandemic period (*P < .*05). The median Brief Resilience Scale score of nurses is 19(6). A negative, weak, and significant relationship was found between the Brief Resilience Scale and Coronavirus Anxiety Scale scores (*P = .*001).

**Conclusion:**

During the pandemic, higher rates of anxiety were detected in healthcare personnel and those working in COVID-19 units. It was also found that as the level of anxiety increased, the level of psychological resilience decreased. To reduce the anxiety level and strengthen the psychological resilience of nurses, the cornerstones of the health system, fast, effective, and curative interventions should be made.

**Main Points**

- The COVID-19 pandemic is a public health problem that has affected the whole world in a short time and caused the death of millions of people.
- COVID-19 has caused high anxiety and burnout in all healthcare professionals, especially nurses.
- As the anxiety level increased, psychological resilience decreased dramatically, and this became even more evident throughout the pandemic.
- Fear of contracting COVID-19 infection caused other segments of society to stay away from healthcare workers, which caused health professionals to experience psychosocial problems.
- The effect of anti-vaccine news on social media and mass media caused a high rate of hesitancy against COVID-19 vaccination among nurses. Despite this, about 80% of the nurses were vaccinated.

## Introduction

On December 31, 2019, some cases of pneumonia of unknown etiology were reported in the city of Wuhan, Hubei province of China. The World Health Organization (WHO) announced on January 9, 2020, that the Chinese authorities determined that this pandemic was caused by a new coronavirus. On February 11, 2020, they named it COVID-19. With the rapid spread of the disease globally, this situation was declared as a pandemic on March 11, 2020.^1,2^ According to the data of WHO, as of April 2022, 500 186 525 confirmed cases and 6 190 349 deaths were detected worldwide.^3^

One of the most important public health issues that were highlighted and extensively discussed during COVID-19 are vaccine hesitancy and anti-vaccination. World Health Organization defines vaccine hesitancy as delay in acceptance or refusing vaccines despite the availability of vaccine services.^4^ Furthermore, it defines anti-vaccination as total opposition to vaccination or advocating against or total refusal of self or one’s child’s vaccination. During the pandemic, healthcare workers were also affected by negative publicity about vaccines and developed hesitancy.^5,6^ This hesitancy of the healthcare workers negatively impacted the public. Therefore, overcoming the hesitancy of healthcare workers will play a crucial role in gaining the trust of the public.

Psychological resilience is defined as the ability to cope with crises, adapt positively, and successfully overcome difficulties.^7^ In a study done in the United States during the pandemic, the participants displayed very low psychological resilience. The study also found a significant link between low psychological resilience and negative psychological conditions such as depression, anxiety, or suicidal tendencies.^8^

Studies have shown that healthcare professionals who work in high-risk and stressful situations during the pandemic are more prone to experience psychological problems such as fear, depression, anxiety, post-traumatic stress symptoms, and insomnia.^9^ A study conducted during the 2003 SARS pandemic showed that nurses and other healthcare professionals who had contact with SARS patients experienced more intense stress.^10^ A study conducted in Spain showed that 71.6% of healthcare professionals working during the COVID-19 pandemic had anxiety symptoms, and 60.3% had depressive symptoms.^11^ In a study conducted on nurses working in a university hospital at the beginning of the pandemic in Turkey, stress, depression, and anxiety levels were found to be significantly higher in nurses.^12^

Healthcare professionals working at critical points during the pandemic may have been professionally exposed to intense physical and psychological stress. For this reason, while the pandemic continues, it is thought that it will be important to determine nurses’ psychological resilience and anxiety levels and develop supportive interventions in this regard. This study aimed to evaluate the vaccine hesitancy, psychological resilience, and anxiety levels of nurses during the COVID-19 pandemic.

## Materials and Methods

### Type, Place, and Time of Research

This survey-based cross-sectional study was conducted between September and October 2021 at Inönü University Turgut Özal Medical Center using the face-to-face interview technique with the staff working as a nurse. Before commencing the study, preliminary permission was obtained from the Director of İnönü University Turgut Özal Medical Center (approval date: August 24, 2021, and number: 77609). Each participant gave verbal consent before the questionnaire was distributed.

### Study Protocol and Ethics Committee Approval

This study involving human participants was in accordance with the ethical standards of the institutional and national research committee and with the 1964 Helsinki Declaration and its later amendments or comparable ethical standards. Ethical approval was obtained from the İnönü University Institutional Review Board for non-interventional studies (approval date: August 24, 2021; number: 2416). Strengthening the reporting of observational studies in epidemiology (STROBE) guideline was utilized to assess the likelihood of bias and overall quality for this study.^13^

### Study Population and Sample Size Calculation

About 900 nurses actively working in the hospital during the abovementioned study period were determined as the population of this study. After entering the confidence level (CL = 95%), confidence interval (CI = 2.5), and patient population (n = 900) data to https://www.surveysystem.com/sscalc.htm to calculate a sample size that can represent this population, the number of samples calculated was determined as 674. A total of 680 nurses were interviewed face-to-face, and 676 nurses who answered all questions were included in this study.

### Variables and Scales Used in the Study

#### Demographic and Social Characteristics Form

The questionnaire used in this study consists of 28 questions and 2 scales. The questions querying the sociodemographic characteristics of the study can be briefly defined as follows: variables such as age, gender, height, weight, marital status, number of children, education level, smoking, presence of chronic disease (diabetes mellitus, hypertension, asthma, chronic obstructive pulmonary disease, cardiovascular disease), presence of psychological disease requiring medication (anxiety, stress, depression), the unit he/she works in the hospital (ward, intensive care, operating room, polyclinics), working status in COVID-19 clinics during the pandemic, catching COVID-19 disease, use of antiviral drugs, hospitalization due to COVID-19 (service, intensive care, intubation), COVID-19 vaccination status (Sinovac, Biontech, both, none), vaccine dose (1, 2, 3, 4 doses), presence of hesitation about the COVID-19 vaccine (hesitant, no-hesitant), belief in the protection of the COVID-19 vaccine, thoughts on making the COVID-19 vaccine legally mandatory, ways to get information about COVID-19 (newspapers, books, magazines, television, social media platforms), post-vaccine COVID-19 disease, and situations causing worry during the COVID-19 process.

#### Coronavirus Anxiety Scale-Short Form

Coronavirus Anxiety Scale, which aims to determine the anxiety caused by the COVID-19 pandemic in society and the severity of this anxiety, was first defined by Lee in 2020.^14^ The validity and reliability test of the Turkish version of this scale was performed by Bicer et al^15^ in 2020 (Cronbach’s alpha = 0.832). The Coronavirus Anxiety Scale consists of 5 questions, and each question is scored between 0 and 4. In the CAS scale consisting of 5-point Likert-type questions, the scores are not at all (0 points), rare, less than a day or 2 (1 point), several days (2 points), more than 7 days (3 points), and nearly every day over the last 2 weeks (4 points). In this scale, where the lowest 0 points and the highest 20 points can be obtained, a score of 9 and above is considered as present with coronavirus anxiety.

### Brief Resilience Scale

Smith et al^16^ developed the Brief Resilience Scale in 2008 to measure the resilience level of individuals. Doğan^17^ performed the validity and reliability test of the Turkish version of this scale in 2015. Brief Resilience Scale, which is a 6-item measurement tool, consists of 5 Likert-type questions, and the answers are listed as strongly disagree (1 point), disagree (2 points), neutral (3 points), agree (4 points), and strongly agree (5 points). Items 2, 4, and 6 on the scale are scored in reverse. Higher scores on the scale indicate higher psychological resilience. Cronbach alpha reliability and internal consistency coefficient of BRS were calculated as 0.830.

### Statistical Analysis

Licensed version 22.0 of the International Business Machines’ Statistical Package for Social Sciences Statistics software program was used for statistical analysis (IBM Corp., Armonk, NY, USA). Shapiro–Wilk test of normality was used to show whether the quantitative variables had normal distribution. Since the continuous variables were observed not to have normal distribution, the results were given as median, and interquartile range (IQR) and 95% CI for the median. Qualitative variables were given as numbers and percentages. Pearson chi-square test was used to compare 2 independent groups. Non-parametric Spearman’s rho correlation analysis was used to show whether there was any correlation between quantitative variables. The *P*-value less than .05 was accepted as significant.

## Results

The median age of the nurses participating in the study was 31 years (IQR: 12; 95% CI: 30-32). 70.7% (n = 478) of the nurses are women, 57.2% (n = 387) are married, and 87.4% (n = 591) have a degree from undergraduate schools. Of the study group, 51.6% (n = 349) stated that they worked in the ward, and 79.6% (n = 538) stated that they did not have a chronic disease. Forty-two percent of the nurses stated that they worked in COVID-19 clinics (ward and intensive care) at least once during the pandemic (Table 1).

A total of 41.4% (n = 280) of the nurses in the study group stated that they had COVID-19, 2.7% (n = 19) of them stated that they were treated in the hospital 1.9% (n = 13) in the ward, 0.7% (n = 5) in intensive care, 0.1% (n = 1) in the ward and intensive care. None of the infected individuals were intubated. A total of 80.9% (n = 547) of the nurses stated that they had the COVID-19 vaccine, 31.5% (n = 213) had the Sinovac vaccine, 19.5% (n = 132) had the BioNTech vaccine, and 29.9% (n = 202) had both. A total of 12.2% (n = 67) of the nurses stated that they had 1 dose of vaccine, 41% (n = 224) of them had 2 doses of vaccine, 43.1% (n = 236) of them had 3 doses of vaccine, 3.7% (n = 20) had 4 doses of vaccine. A total of 68.6% (n = 464) of the nurses participating in the study stated that they were hesitant about the COVID-19 vaccine, 50.0% (n = 338) thought that this vaccine was protective, and 27.5% (n = 186) were undecided about the protectiveness of the vaccine. A total of 51.2% (n = 346) of the participants stated that they had read scientific articles about COVID-19 and vaccination. When asked about their sources of information on COVID-19 and vaccination, 63.2% (n = 427) of nurses stated that the source of information was newspapers, books, magazines, or articles, 62.9% (n = 425) stated that they obtained information from social media, 56.8% (n = 384) from health programs on television, and 37.9% (n = 256) from their relatives or neighbors who had the disease. When nurses were asked about the most worrisome situation(s) during the COVID-19 period, the highest response rates were 82.2% (n = 556) for the parents’ catching COVID-19 and 47.5% (n = 321) for the unknowns about COVID-19 being high (Table 2).

68.6% (n = 464) of the participants stated they were hesitant about the COVID-19 vaccine. A significantly higher rate of hesitancy was detected in the age group of 20-29 years and 30-39 years, those who did not have COVID-19 vaccine, and those who did not think the COVID-19 vaccine is protective (*P < .*05). There was no significant difference between COVID-19 vaccine hesitancy according to gender and education level (*P* > .05) (Table 3)

It was determined that 6.8% (n = 46) of the nurses had COVID-19 anxiety. There was no significant difference between the presence of COVID-19 anxiety and gender and the presence of chronic disease (*P* > .05). A significantly higher rate of anxiety was detected in the age group of 40 years and older, those working in the emergency department, and those working in the COVID-19 unit during the pandemic period (*P < .*05). No significant difference was found between the presence of COVID-19 anxiety according to the COVID-19 status (*P = .*546) (Table 4).

The median BRS score of nurses is 19 (IQR = 6). A negative, weak, and significant relationship was found between the BRS and the CAS scores. The CAS score decreases as the BRS score increases (Table 5).

## Discussion

Half of the nurses participating in our study stated that the COVID-19 vaccine is protective, 27.5% are undecided about the vaccine’s protection, 80.9% have the COVID-19 vaccine, and 68.6% are hesitant about the COVID-19 vaccine. In a study conducted on healthcare professionals in France, it was shown that 23.1% (n = 453) of the participants were hesitant about COVID-19 vaccines, and 3.9% (n = 76) were anti-COVID-19 vaccines.^18^ In a study conducted among 1723 healthcare professionals in Italy, it was stated that 67% (n = 1155) of the participants were willing to be vaccinated against COVID-19, 26% (n = 443) were undecided, and 7% (n = 125) refused to be vaccinated.^19^ In a study conducted with healthcare professionals in Egypt, it was shown that 41.9 % (n = 129) of the participants were undecided, 32.1% (n = 99) refused, and 26% (n = 80) were willing to COVID-19 vaccines.^20^ A study conducted with healthcare professionals in Canada showed that 31.5% (n = 84) of those who were hesitant about COVID-19 vaccines thought that the vaccine was not protective.^21^ A study conducted with nurses in China in 2020 showed that 76.4% (n = 360) of the participants had doubts about the efficacy or safety of the vaccine.^22^ Healthcare professionals, who are the first group to be vaccinated, can be important role models for society, as they are generally the priority group around the world. Attitudes and behaviors of healthcare professionals about vaccination may affect the vaccination decisions of hesitant individuals. For this reason, it will be important to address the hesitations of healthcare professionals about vaccines and underlying causes and concerns, to conduct studies in this direction, and to provide more information about the safety and effectiveness of vaccines.

About 51.2% of the nurses participating in our study stated that they read scientific articles about COVID-19 and vaccination, and 63.2% stated that the source of information about COVID-19 and vaccination is in newspapers, books, magazines, or articles. A study conducted with healthcare professionals in Italy has shown that nurses use websites, social media, television, newspaper, family, and friend suggestions more as a source of information about COVID-19, and the rate of using scientific literature is lower.^23^ A study conducted with healthcare professionals in Egypt has shown that 85% (n = 68) of the participants who were willing to be vaccinated against COVID-19 used the websites of WHO and the Center for Disease Control and Prevention (CDC) as a source of information about the vaccine.^20^ Healthcare professionals should have sufficient and up-to-date scientific knowledge about pandemics and vaccines. Considering the increasing vaccine rejection and hesitation in society, the information resources of healthcare professionals stand out. Therefore, it is important for nurses, who are healthcare personnel, to obtain the correct information from the right source.

It was stated that the most worrying situation for nurses during the COVID-19 period was parents catching COVID-19 and high uncertainty about COVID-19. Despite the highest protection measures, healthcare professionals may be at high risk of catching COVID-19 during the pandemic. For this reason, the high mortality and morbidity rates, especially in older parents, may have created this concern.

The vaccine hesitancy among nurses in this study was higher (68.6%) than expected. In another study, vaccine hesitancy rate was found to be 25.9% (n = 531)^24^ among healthcare workers, while 2 different studies done on the general public showed hesitancy rates of 36% (n = 540)^25^ and 35.9% (n = 1098).^26^ As anticipated, the vaccine hesitancy rates were lower among nurses who believed COVID-19 to be beneficial and those that had already received a vaccination.

The health belief model is a behavioral change model developed to explain the decision-making processes about human health and resulting behavioral changes. In this model, perceived sensitivity contributes to promoting decision-making based on various perceived stimulus such as benefits and barriers.^27^ Jain et al^28^ stated that the perceived susceptibility to COVID-19 and the perceived benefits of vaccination had a crucial role in reducing hesitancy for COVID-19 vaccination. They stated that these findings match the health belief model. Similarly, in this study, the thought that the COVID-19 vaccines are safe and beneficial could have contributed to lower hesitancy rates among the vaccinated nurses who believed that the vaccines were protective. 

In our study, it was determined that 6.8% of the participants had COVID-19 anxiety, and a significantly higher rate of anxiety was detected in those working in the COVID-19 unit, in the group over the age of 40, and in the emergency room during the pandemic period. In a study conducted in Italy, it was shown that stress (*P = .*013), exhaustion (*P = .*037), anxiety (*P = .*014), and depression (*P = .*013) were higher in healthcare professionals dealing with COVID-19 patients.^29^ In a study conducted in Oman, it was shown that anxiety (odds ratio (OR) = 1.557, *P = .*004), stress (OR = 1.506, *P = .*016), and insomnia (OR = 1.586, *P = .*013) states of healthcare professionals working on the front lines in the COVID-19 pandemic were 1.5 times more common than those in the other group.^30^ In a study conducted on healthcare professionals in China, depression and anxiety were found in 50.4% (n = 634) and 44.6% (n = 560) of the participants, respectively, and similar to our study, it was shown that professionals working in the COVID-19 unit experienced more severe depression and anxiety.^31^

It was determined that the level of psychological resilience decreased as the COVID-19 anxiety level of the study group increased. In a study conducted with healthcare professionals in Spain during the first wave of the pandemic, it was shown that nurses and assistant nurses experienced mental disorders at a higher rate, and a positive trend was found in those whose parents, children, or partners were infected by COVID-19.^32^ As shown in other studies similar to our study, in the healthcare professionals working on the front lines of the pandemic, higher levels of anxiety and depression were observed in the vaccine group of older age, with the thought that the age group increases the risk, and it is thought that taking precautions and interventions accordingly may have a healing effect in terms of mental health and work efficiency of healthcare professionals.

Considering that new pandemics may occur with new infectious agents in the coming years, urgent measures should be taken against anxiety, depression, and exhaustion in healthcare personnel. It is thought that additional interventions such as increasing personnel employment, arranging working shifts, and psychosocial support should be made.

### Limitations

As in all other survey-based studies, this study has some limitations. It is very difficult to make a comment on the generalization of the results to the universe since the entire universe cannot be reached. Among the reasons for this situation are the shift work system, intensive working conditions, and the official leave of those with chronic diseases.

## Conclusion

To sum up, necessary precautions should be taken to prevent anxiety, depression, and other psychological conditions that may occur in nurses who are active healthcare personnel during the pandemic period, and the awareness of healthcare personnel on this issue should be increased.

## Figures and Tables

**Table 1.** Distribution of Sociodemographic and Other Variables of the Nurses Participating in the Study

Variables of the Participants	n	%
**Age groups (year)**		
20-29	278	42.9
30-39	246	38.0
≥ 40	124	19.1
**Gender**		
Female	478	70.7
Male	198	29.3
**Marital status**		
Married	387	57.2
Single	289	42.8
**Educational status**		
High school/Assoc graduate	42	6.2
Bachelor’s degree	591	87.4
Master’s/Doctorate	43	6.4
**Working unit**		
Service (wards)	349	51.6
Intensive care	248	36.7
Emergency unit	13	1.9
Operating room	29	4.3
Outpatient clinic	37	5.5
**Did you have chronic disease?**		
Yes	138	20.4
No	538	79.6
**Did you work in COVID-19 units?**		
Yes	284	42.0
No	392	58.0
**Exposure to the COVID-19**		
Yes	280	41.4
No	396	58.6
**Brief Resilience Scale Score**		
Median (IQR)	19 (6)	
95% CI for median	19-20	
**Coronavirus Anxiety Scale Score**		
Median (IQR)	1 (3)	
95% CI for median	1-1	

IQR, interquartile range.

**Table 2.** Distribution of COVID-19 Vaccine Hesitancy and Various Variables of Nurses Participating in the Study

Variables of the Participants	n	%
**Vaccinated against COVID-19?**		
Yes	547	80.9
No	129	19.1
**Number of COVID-19 vaccines **		
1 dose	67	12.2
2 dose	224	41.0
3 doses	236	43.1
4 doses	20	3.7
**Hesitancy against COVID-19 vaccine**		
Hesitant	464	68.6
No-Hesitant	212	31.4
**Do you think the COVID-19 vaccine is protective?**		
Yes	338	50.0
No	152	22.5
No idea	186	27.5
**Have you read scientific articles about COVID-19 and vaccine?**		
Yes	346	51.2
No	152	22.5
**Which is your source of information about COVID-19 and vaccination?**		
From newspapers, books, magazines, or articles	427	63.2
Social media (Whatsapp, Facebook, Instagram, etc.)	425	62.9
Health programs on television	384	56.8
Relatives or neighbors who have had the disease	256	37.9
Other		
**Which of the following worries you the most, during COVID-19 era durum/durumlar**		
My parents’ exposure to COVID-19	556	82.2
Uncertainties about COVID-19	321	47.5
Individual exposure to COVID-19	223	33.0
Working in the COVID-19 intensive care unit	109	16.1
Working in the COVID-19 service	96	14.2
**Should the COVID-19 vaccine be made mandatory by law? **		
Yes	219	32.4
No	324	47.9
No idea	133	19.7

**Table 3.** Comparison of the COVID-19 Vaccine Hesitancy Status of the Nurses Participating in the Study According to Various Variables

Variables of the Participants	COVID-19 Vaccine Hesitancy Status				*P*
	Hesitant (n = 464)		No-Hesitant (n = 212)		
	n	%	n	%	
**Age groups (year)**					
20-29	202	72.7	76	27.3	**.035**
30-39	169	68.7	77	31.3	
≥40	74	59.7	50	40.3	
**Gender**					
Female	334	69.9	144	30.1	.282
Male	130	65.7	68	34.3	
**Educational status**					
High school/Assoc graduate	29	69.0	13	31.0	.835
Bachelor’s degree	408	69.0	183	31.0	
Master’s/Doctorate	26	63.4	15	36.6	
**Do you think the COVID-19 vaccine is protective?**					
Yes	182	53.8	156	46.2	**<.001**
No	132	86.8	20	13.2	
No idea	150	80.6	36	19.4	
**Have you had the COVID-19 vaccine?**					
Yes	347	63.4	200	36.6	**<.001**
No	117	90.7	12	9.3	

**Table 4.** Comparison of the Coronavirus Anxiety Status of the Nurses Participating in the Study According to Various Variables

Variables	COVID-19 Anxiety Status				*P*
	Presence (n = 46)		Absence (n = 630)		
	n	%	n	%	
**Age groups (year)**					
20-29	13	4.7	265	95.3	**.051**
30-39	17	6.9	229	93.1	
≥40	14	11.3	110	88.7	
**Gender**					
Female	35	7.3	443	92.7	.407
Male	11	5.6	187	94.4	
**Did you have chronic disease?**					
Yes	11	8.0	127	92.0	.542
No	35	6.5	503	93.5	
**Working Unit**					
Service (wards)	24	6.9	325	93.1	**.013**
Intensive care	15	6.0	233	94.0	
Emergency unit	4	30.8	9	69.2	
Operating room	1	3.4	28	96.6	
Outpatient clinic	2	5.4	35	94.6	
**Did you work in COVID-19 Units?**					
Yes	14	14.6	82	85.4	**.001**
No	32	5.5	548	94.5	
**Have you been exposed to COVID-19?**					
Yes	21	7.5	259	92.5	.546
No	25	6.3	371	93.2	

Presence (score ≥ 9 point), Absence (score < 9).

**Table 5.** Correlation of the CAS and BRS Scores of the Nurses Participating in the Study

Brief Resilience Scale	Coronavirus Anxiety Scale
***r***	**−0.315**
***P***	**<.001**
**n**	**657**

BRS, Brief Resilience Scale; CAS, Coronavirus Anxiety Scale.
